# Scalable Sub-micron Patterning of Organic Materials Toward High Density Soft Electronics

**DOI:** 10.1038/srep14520

**Published:** 2015-09-28

**Authors:** Jaekyun Kim, Myung-Gil Kim, Jaehyun Kim, Sangho Jo, Jingu Kang, Jeong-Wan Jo, Woobin Lee, Chahwan Hwang, Juhyuk Moon, Lin Yang, Yun-Hi Kim, Yong-Young Noh, Jae Yun Jaung, Yong-Hoon Kim, Sung Kyu Park

**Affiliations:** 1 School of Electrical and Electronic Engineering, Chung-Ang University, Seoul, Korea; 2 Department of Applied Materials Engineering, Hanbat National University, Daejeon, Korea; 3 Department of Chemistry, Chung-Ang University, Seoul, Korea; 4 School of Advanced Materials Science and Engineering, Sungkyunkwan University, Suwon, Korea; 5 SKKU Advanced Institute of Nanotechnology (SAINT), Sungkyunkwan University, Suwon, Korea; 6 Civil Engineering Program, Department of Mechanical Engineering, Stony Brook University, NY, USA; 7 Photon Sciences Directorate, Brookhaven National Laboratory, Upton, NY, USA; 8 Department of Chemistry Gyeongsang National University and Research Institute of Nature Science (RINS), Jinju, Korea; 9 Department of Energy and Materials Engineering, Dongguk University, Seoul, Korea; 10 Department of Organic and Nano Engineering, Hangyang University, Seoul, Korea

## Abstract

The success of silicon based high density integrated circuits ignited explosive expansion of microelectronics. Although the inorganic semiconductors have shown superior carrier mobilities for conventional high speed switching devices, the emergence of unconventional applications, such as flexible electronics, highly sensitive photosensors, large area sensor array, and tailored optoelectronics, brought intensive research on next generation electronic materials. The rationally designed multifunctional soft electronic materials, organic and carbon-based semiconductors, are demonstrated with low-cost solution process, exceptional mechanical stability, and on-demand optoelectronic properties. Unfortunately, the industrial implementation of the soft electronic materials has been hindered due to lack of scalable fine-patterning methods. In this report, we demonstrated facile general route for high throughput sub-micron patterning of soft materials, using spatially selective deep-ultraviolet irradiation. For organic and carbon-based materials, the highly energetic photons (e.g. deep-ultraviolet rays) enable direct photo-conversion from conducting/semiconducting to insulating state through molecular dissociation and disordering with spatial resolution down to a sub-μm-scale. The successful demonstration of organic semiconductor circuitry promise our result proliferate industrial adoption of soft materials for next generation electronics.

Recently developed high-performance organic and carbon-based materials have demonstrated charge carrier mobilities and conductivities greater than 10 cm^2^ V^−1^ s^−1^ 1,2,3,4 and 10^3^–10^4^ S cm^−1^ 5,6,7, respectively, which are superior to those of industrial standard materials such as amorphous silicon and indium-tin-oxide. Although the outstanding electrical properties of such soft materials position them as promising building blocks for next-generation flexible electronics, reliable and scalable fine-patterning technology should also be accompanied for the realization of high-density and multi-functional soft electronics. Typically, proper isolation/patterning of the functional materials is required to suppress parasitic and off current, leading to less cross-talk between neighboring devices and minimum power consumption in high-density integrated systems^8^,^9^. Fluorinated photoresists using an orthogonal solvent^10^, photochemical dimerization of specific organic molecule^11^, pre-patterned self-assembled monolayers^12^,^13^, and various direct printing techniques^14^ have been used to pattern such soft materials; however, several drawbacks, such as the process complexity, limited choice of materials, low throughput, and the resolution limit, have proven problematic for industrial realization. Mainly based on photo-oxidation, the photobleaching of light emitting materials have been applied for the high-resolution patterning of organic light emitting diode (OLED)^15^,^16^. Although the photobleaching process was quite successful for the monochromatic OLED device patterning, the approach was only demonstrated for controlling fluorescence of π-conjugated materials. Here, we report a facile and general route to achieve scalable high-resolution (sub-micron) patterning of organic and carbon-based materials for device and material integrations via photochemically induced molecular disordering. Upon deep-ultraviolet (DUV) irradiation, the soft matters experienced dissociation of specific chemical bonds within molecules as well as the loss of inter-molecular ordering, transforming them into a non-functional state. Spatially selective DUV irradiation enables large arrays of patterned functional devices on a substrate. Various organic and carbon-based thin-film-transistors were fabricated using this patterning approach; the resulting transistors exhibited well-defined active material isolation (current on/off ratio: >10^7^) and minimized parasitic current (on the order of pA). The transistors were used to fabricate low-power consumption integrated circuits on both rigid and flexible substrates without compromising their individual device performance.

## Result and Discussion

**Figure 1:**
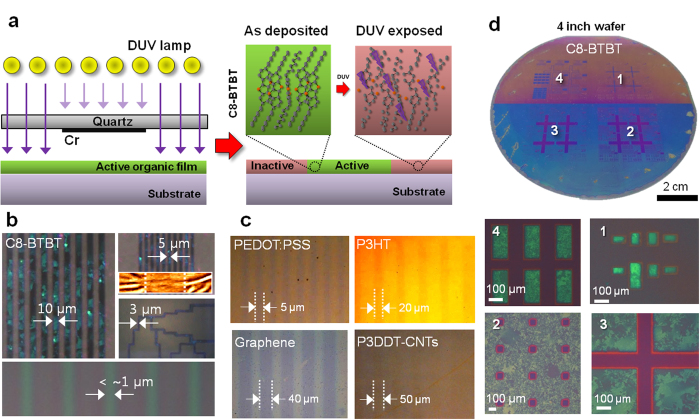
DUV patterning of organic thin films. (**a)** Schematic diagram of the DUV exposure system and the resulting photochemical modification, illustrating the chemical bond dissociation and loss of molecular ordering of a C8-BTBT small molecule from the exposed region as an example. (**b**) CPOM images of C8-BTBT line patterns with a width of 10 and 5 μm, clearly showing a strong birefringence from the unexposed region. 3-μm wide complex pattern and 1-μm line width were also defined by DUV irradiation. AFM surface scan exhibits the difference of C8-BTBT morphology upon DUV irradiation. (**c**) Patterned PEDOT:PSS, P3HT, graphene, and P3DDT-wrapped CNT films exhibiting a periodic optical contrast. White dashed lines are inserted as a guide for the eye and indicate mask-protected regions. Note that excimer lamp was used for the graphene while low-pressure mercury lamp for other soft materials. (**d**) Large-area DUV patterning performed on a 4-inch wafer; four-quadrant CPOM shows the uniformity of patterning.

Room-temperature photochemical routes via deep-ultraviolet (DUV) irradiation have been known to be effective in cleaving specific chemical bonds^17^,^18^,^19^,^20^, which inspired us to explore the possibility of the chemical-free fine-patterning of organic and carbon-based functional materials using high-energy photon irradiation. Figure 1a shows a schematic of the direct photochemical patterning of the soft materials via DUV exposure through a quartz photomask. The high-energy photons, from low pressure mercury lamp (LPML) [90% 4.88 eV (254 nm) and 10% 6.70 eV (185 nm)] or radio frequency (RF) discharge excimer lamp [7.21 eV (172 nm)], exceed the typical dissociation energies of the molecular bonds of soft materials, such as the dissociation energies of C–S (2.69 eV), C–C (3.65 eV), C–H (4.25 eV), and C **=** C (6.32 eV)^21^. In organic and carbon-based electronic materials, this DUV irradiation can be expected to induce chemical bond dissociation and the subsequent loss of carrier transport mechanisms, such as molecular packing for π-π interactions^22^, crystalline domain interconnections^23^, and electron delocalization within extended carbon materials^24^. Although we typically applied low cost LPML irradiation for chemically weak organic semiconductors, the strong internal chemical bonding in carbon materials requires high energy excimer lamp irradiation with residual oxygen. Figure 1b shows the cross-polarized optical microscopy (CPOM) images of DUV-patterned 2,7-dioctyl[1]benzothieno[3,2-b][1]benzothiophene (C8-BTBT) line arrays on a silicon substrate with an exposure time of 30 min. The C8-BTBT films in unexposed areas exhibit strong birefringence, which is indicative of molecular ordering, whereas DUV-irradiated regions don’t respond to the polarized light (Supplementary Fig. 1). This result suggests that the crystalline molecular structures change into an amorphous phase via the photochemical deactivation route. Fine patterning of the C8-BTBT organic films to sub-micron linewidths is also shown in the bottom of Fig. 1b. As shown in Fig. 1c, this photochemical route can be applied to various organic and carbon-based materials, including poly(3-hexylthiophene) (P3HT), poly(3,4-ethylenedioxythiophene) poly(styrenesulfonate) (PEDOT:PSS), graphene and poly(3-dodecylthiophene) (P3DDT)-wrapped carbon nanotube (CNT) films, thereby creating a periodic change of contrast. The full utilization of this approach at the device and system levels would be greatly facilitated by the development of a scalable fabrication method that overcomes the resolution limitation while providing uniform patterned structures. Figure 1d shows a photograph of a DUV-patterned C8-BTBT film on a 4-inch silicon wafer. The residue-free and uniformly developed organic patterns over the entire wafer are clearly demonstrated in the four-quadrant high-magnification CPOM images in Fig. 1d.

**Figure 2:**
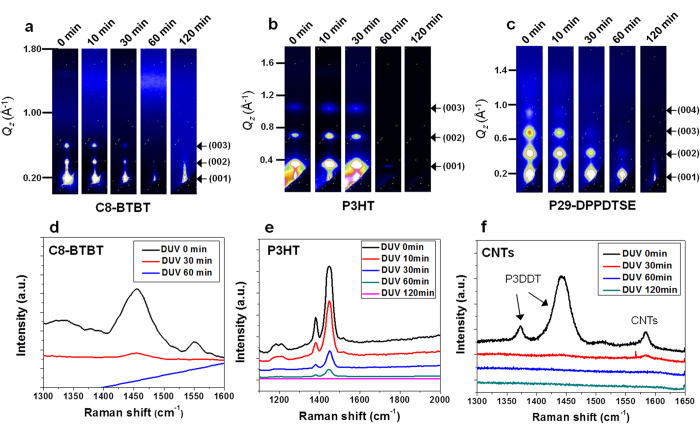
Intermolecular disordering and chemical-bond dissociation of DUV-exposed small molecule, polymer, and carbon-based films. GIXRD patterns of Bragg diffraction peaks of (**a**), C8-BTBT (small molecule), (**b**) P3HT (polymer), and (**c**) P-29-DPPDTSE (high-performance polymer) in the *Q*_z_ direction (reciprocal space) as a function of DUV irradiation time. Gradual decreases in the intensity of Bragg diffraction peaks are clearly visible with increased DUV exposure time. Raman spectra of (**d**) C8-BTBT, (**e**) P3HT, and (**f**) P3DDT-wrapped CNTs, indicating the disappearance of organic-related characteristic peaks after DUV irradiation.

Grazing incidence X-ray diffraction (GIXRD) profiles of the organic semiconductor films were collected to investigate the effect of DUV irradiation on the high degree of inter-molecular ordering within organic semiconductors. As shown in Fig. 2a–c, the as-prepared films of C8-BTBT and P3HT and poly-[2,5-bis(2-decyltetradecyl)pyrrolo[3]pyrrole-1, 4-(2*H*,5*H*)-dione-(*E*)-(1,2-bis(5-(thiophen-2-yl)selenophen-2-yl)ethene] (P-29-DPPDTSE)^2^ showed apparent out-of-plane Bragg diffraction peaks at each Q_z_ vector; these peaks represent the well-ordered alkyl chain stacking distance normal to the substrate. With increasing DUV irradiation time, the diffraction peaks obviously decreased in intensity and finally disappeared. Complete removal of inter-molecular ordering from all organic films was observed after 60 min of continuous DUV irradiation. A gradual decrease in the intensity of the in-plane diffraction peaks with increased DUV irradiation confirmed the isotropic degradation of molecular ordering within the organic semiconductors, consistent with the CPOM results (Supplementary Figs 2 and 3).

Raman spectroscopic curves for the diverse organic films and P3DDT-wrapped CNT used in this study are shown in Fig. 2d–f and Supplementary Fig. 4, respectively; these spectra indicate universal chemical bond rupture in the materials. In Fig. 2d,e, the Raman spectra of C8-BTBT and P3HT exhibit clear decreases in the intensities of thiophene-related peaks as a result of high-energy photon influx^25^,^26^. Upon DUV irradiation of the graphene (excimer lamp) and P3DDT-wrapped CNT (LPML), the G-peaks at ~1580–1590 cm^−1^ in the graphene and CNT Raman spectra (Supplementary Fig. 4e and Fig. 2f) abruptly decreased in intensity; these peaks disappeared completely. Identical chemical bond ruptures were generally observed in most of the DUV-irradiated materials, as shown in Supplementary Fig. 4. Moreover, the molecular-level degradation of the soft materials resulted in morphological changes on their surfaces, as shown in the AFM images in Supplementary Fig. 5. The reduction of surface roughness represents flattening of the crystalline domain of small molecules/polymers. The clear connection between molecular-level spectroscopy with inter- and intra-molecular ordering, as revealed by AFM imaging and GIXRD experiments, indicates that the incidence of high-energy photons to the soft materials severely disrupts the molecular packing, crystalline domain interconnections, and delocalized chemical bonds, which are essential for efficient charge transport in these materials.

**Figure 3:**
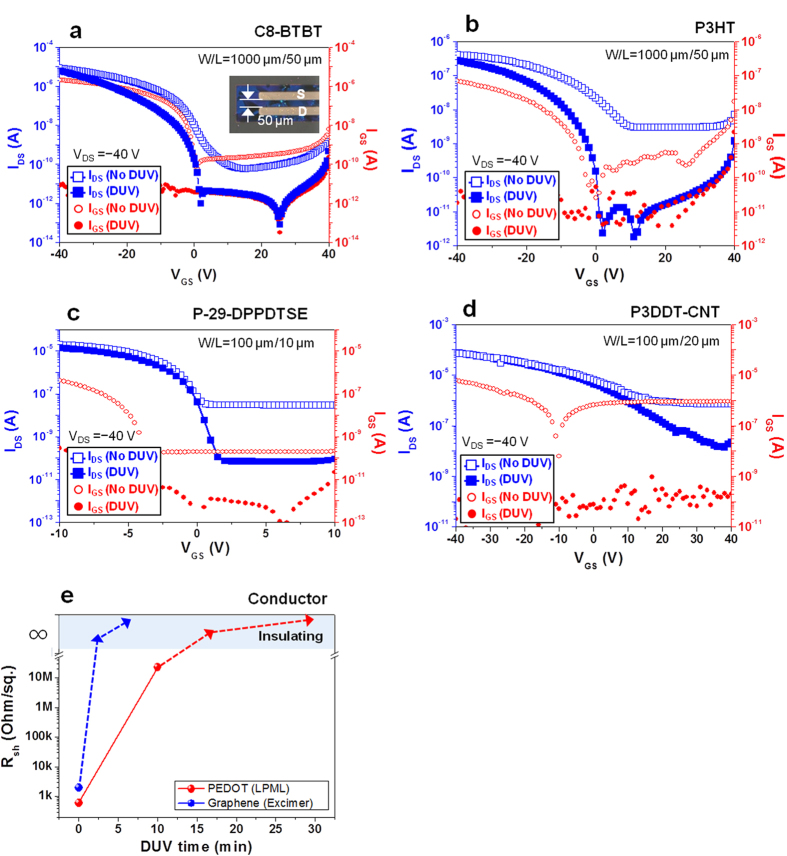
Electrical characterization of organic-based transistors and conductors upon DUV irradiation. Comparison of transfer curves of OTFT-based on (**a**) C8-BTBT, (**b**) P3HT, (**c**) P-29-DPPDTSE, and (**d**) P3DDT-CNT films before and after DUV active region isolation; the curves exhibit an excellent drain-current modulation (high on/off ratio as large as ~10^7^) by a gate bias and minimized parasitic current (I_GS_). CPOM image of isolated C8-BTBT OTFT is inserted as inset in (**a**). (**e**) Sheet resistance change of organic and carbon-based conductors with increased LPML and excimer lamp irradiation time, respectively. Excimer lamp irradiation with shorter wavelength and higher intensity significantly reduces the process time.

Various organic and CNT thin-film transistors (OTFTs and CNT TFTs, respectively) were fabricated by active layer isolation to demonstrate the fidelity of the mechanism in real thin-film device applications. CPOM images of small-molecule-based OTFTs clearly indicate the isolated channel region (Supplementary Fig. 6 and the inset of Fig. 3a). Figure 3a–d and Supplementary Fig. 7 clearly show improvements in the transfer curves of these soft-material-based TFTs after their semiconducting layer was isolated via spatially selective DUV exposure. Without proper isolation of the semiconducting layers, a significant gate current (I_GS_, roughly one-tenth of the drain current (I_DS_)) and relatively low current on/off ratios (~10^3^) were observed in most of the devices. These undesired/parasitic currents are often associated with cross-talk between adjacent devices in high-density integrated circuits, which becomes increasingly problematic when device dimensions are downscaled. Apparently, photochemical isolation enables a substantial decrease of I_GS_ by more than 4 orders of magnitude while simultaneously decreasing the off-state I_DS_ to as low as the sub-nA level (see the red symbols and lines in the transfer curves of Fig. 3a–d and Supplementary Fig. 7), which are almost identical to the physical isolation cases. Undoubtedly, DUV-exposed device exhibits a negligible I_DS_ as a consequence of photochemical degradation of organic film (Supplementary Fig. 8). From the electrical data (Fig. 3 and Supplementary Fig. 7) and GIAXRD patterns (Fig. 2a–c and Supplementary Figs 2 and 3), typically ~72 J/cm^2^ UV dose (30 min exposure with 20 mW/cm^2^ intensity LPML DUV irradiation) is required to completely destroy the charge transport in organic semiconductors. The high UV dose requirements were also reported on complete photochemical reaction of relatively poor UV sensitive organic materials^27^,^28^. These series of transfer and output (Supplementary Figs 9 and 10 for small molecules and polymers) curves before and after DUV isolation demonstrate clear evidence of effective device isolation for organic and carbon-based semiconductors with unprecedented simplicity. Narrowing down the channel width (W) of organic transistors down to 2 μm revealed the gradual decrease of I_DS_, which seemed to be quite consistent with their theoretical relation (I_DS_ ∝ W). So, it can be claimed that further downscaling for smaller dimension of organic-patterned devices could be achievable with our chemical-free DUV irradiation (Supplementary Fig. 11). In addition to electrical isolation, the field-effect mobility must remain unimpaired during DUV exposure for successful device isolation. For instance, as evident from the I_DS_ of P-29-DPPDTSE OTFTs at the saturation regime in Supplementary Fig. 7a, the small field-effect mobility (μ_sat_) difference between as-fabricated (2.75 cm^2^ V^−1^ s^−1^) and DUV-isolated (2.71 cm^2^ V^−1^ s^−1^) devices indicates no noticeable device degradation, even when the fringing current is excluded. Similarly, negligible performance degradations were observed in the cases of other organic semiconductors and carbon materials after the active-channel isolation procedure. Electrical parameters, such as μ_sat_ and V_TH_, for various TFTs based on the aforementioned organic and carbon-based materials are summarized in Supplementary Table S1.

Additionally, the difficulty associated with fine patterning in conductive materials has impeded engineering applications of these soft materials. In addition to fine patterning of PEDOT:PSS, and graphene films, the sheet resistances of all conducting films were modulated from about ~10^2^ to ~10^7^ Ohm/square, as measured by a 4-point contact probe, after 1–10 min of DUV irradiation, as shown in Fig. 3e. Further DUV irradiation resulted in an insulating film with an immeasurably low sheet resistance. Thus, the photochemical deactivation of soft material films could provide a simple and effective patterning route for the conductive materials and could be applicable to newly developed two dimensional semiconducting or conducting materials^29^.

**Figure 4:**
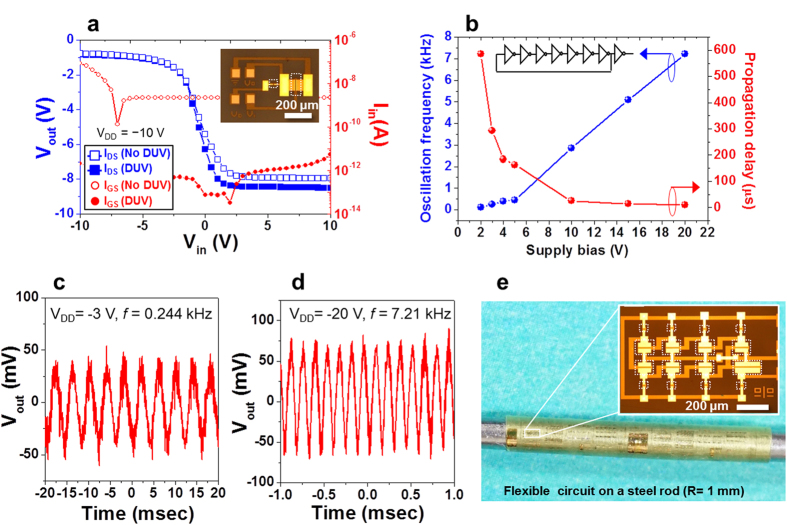
Flexible OTFT-based integrated circuits with low power consumption. (**a**) Electrical characteristics of an inverter based on P-29-DPPDTSE OTFTs after DUV isolation, highlighting the change in the input current (I_in_). (**b**) Plot of oscillation frequency and per stage propagation delay of 7-stage ring oscillator fabricated on a 3-μm-thick PI film as a function of supply voltage (V_DD_). (**c**) and (**d**) Output waveforms of the ring oscillator operating from supply voltages of −3 V and −20 V, exhibiting 0.244 kHz and 7.21 kHz, respectively. (**e**) Photograph and OM images of OTFT-based flexible integrated circuits that conformably wraps around the steel rod (R = 1 mm). The dashed white boxes in (**a**) and (**e)** indicate the chrome patterns of photomask protecting the active region during DUV exposure.

The off-state current is often considered as an origin of static power consumption in most integrated circuits^30^, as well as a source of contrast degradation in displays^8^. Without device isolation, power consumption for long-term operation of these systems could be multiplied by off-state current. Flexible P-29-DPPDTSE OTFT devices were also isolated using DUV exposure following fabrication of the devices on an ultrathin (3-μm-thick) polyimide substrate (Supplementary Fig. 12). Figure 4a shows a simple inverter circuit and its characteristics measured from P-29-DPPDTSE OTFTs with a β ratio of 10 on a PI film before and after DUV isolation. We observed an approximately 3 orders of magnitude smaller supply current (I_in_) and noticeable reduction of supply current (I_DD_) while maintaining almost identical or enhanced gain in the inverter by active layer isolation via DUV irradiation (Supplementary Fig. 13). Figure 4b shows a relation of the oscillation frequency and the single-stage propagation delay as a function of supply voltage, exhibiting an oscillation frequency up to 7.21 kHz at V_DD_ = −20 V. The corresponding output waveforms at the low and high supply voltage of V_DD_ = −3 V and −20 V are shown in Fig. 4c,d, respectively. Figure 4e shows the flexible high-performance organic circuits wrapping around the steel rod (R = 1 mm), highlighting the successful isolation of devices packed at fairly high density (approximately 3.8 × 10^3^ cm^−2^). Additionally, based on an AIM-SPICE simulation, the power consumption of a 7-stage ring oscillator can be reduced by about 20% after the organic channel isolation due to the reduction of leakage current (Supplementary Fig. 14).

In summary, we demonstrated that spatially selective DUV irradiation can enable high-density fine pattern formation for a wide range of organic and carbon-based films, including small molecules, polymers, CNT, and graphene. This single-step, chemical-free DUV deactivation takes advantage of atomic-level bond dissociation and loss of inter-molecular ordering within the soft functional materials. Therefore, we envision that this selective DUV irradiation could offer an innovative scientific and technological approach to the low-cost and large-volume patterning of high-density organic and carbon-based electronics with excellent uniformity and resolution.

## Methods

We prepared the organic and carbon-based films using simple solution processes (see the supplementary information for details). For the LPML DUV irradiation, a DUV generator (SAMCO UV-1) equipped with a low-pressure mercury lamp, which emitted main peaks at 253.7 nm (90%) and 184.9 nm (10%), was used; the radiation intensity was 18–23 mW cm^−2^. For the 172 nm high energy irradiation, RF discharge excimer lamp (Hamamatsu Photonics K.K. EX-mini L12530) was used; the radiation intensity was 50 mW cm^−2^. The DUV irradiations were performed with chrome-patterned quartz masks under N_2_ atmosphere.

The crystalline nature of DUV-patterned C8-BTBT small-molecule organic films was visualized using cross-polarized optical microscopy. Other patterned films of soft materials, including P3HT, PEDOT:PSS, and graphene were visualized using optical microscopy. Grazing incidence wide-angle X-ray scattering (GIWAX) measurements were performed at the X9 beamline of the National Synchrotron Light Source at Brookhaven National Laboratory. Surface morphologies of organic films before and after DUV irradiation were examined using AFM system in non-contact mode. Raman spectra of organic films were obtained with a confocal Raman spectroscopy system.

For the fabrication of various OTFTs, organic semiconductor films were contacted by 50-nm-thick thermally evaporated Au electrodes on a heavily doped silicon wafer (gate) bearing a 200-nm thermal oxide. For individually addressable OTFTs and circuit fabrication, a 50-nm-thick sputtered Cr film as a gate electrode on a glass substrate and on a PI film were patterned by conventional photolithography. 35-nm-thick Al_2_O_3_ as a gate insulator was vacuum-deposited using atomic layer deposition system at 100 °C. Source and drain electrodes with Cr/Au (3/50 nm) were formed by a lift-off process. All devices and circuits measurements were performed with an Agilent 4156 C semiconductor parameter analyzer under dark and ambient-air conditions.

## Additional Information

**How to cite this article**: Kim, J. *et al.* Scalable Sub-micron Patterning of Organic Materials Toward High Density Soft Electronics. *Sci. Rep.* **5**, 14520; doi: 10.1038/srep14520 (2015).

## Supplementary Material

Supplementary Information
