# Supplementary material for: Scalable Sub-micron Patterning of Organic Materials Toward High Density Soft Electronics
Source: Sci Rep. 2015 Sep 28;5:14520. doi: 10.1038/srep14520 (PMC4585943; doi:10.1038/srep14520)
Supplement: Supplementary Information [file srep14520-s1.doc]

Supplementary Information

Scalable Sub-micron Patterning of Organic Materials

Toward High Density Soft Electronics

Jaekyun Kim^1,2^†, Myung-Gil Kim^3^†, Jaehyun Kim^1^, Sangho Jo^1^, Jingu Kang^1^, Jeong-Wan Jo^1^, Woobin Lee^4,5^, Chahwan Hwang^3^, Juhyuk Moon^6^, Lin Yang^7^, Yun-Hi Kim^8^, Yong-Young Noh^9^, Jae Yun Jaung^10^, Yong-Hoon Kim^4,5^, and Sung Kyu Park^1^*

^1^ School of Electrical and Electronic Engineering, Chung-Ang University, Seoul, Korea

^2^ Department of Applied Materials Engineering, Hanbat National University, Daejeon, Korea

^3^ Department of Chemistry, Chung-Ang University, Seoul, Korea

^4^ School of Advanced Materials Science and Engineering, Sungkyunkwan University, Suwon, Korea

^5^ SKKU Advanced Institute of Nanotechnology (SAINT), Sungkyunkwan University, Suwon, Korea

^6^ Civil Engineering Program, Department of Mechanical Engineering, Stony Brook University, NY, USA

^7^ Photon Sciences Directorate, Brookhaven National Laboratory, Upton, NY, USA

^8^ Department of Chemistry Gyeongsang National University and Research Institute of Nature Science (RINS), Jinju, Korea

^9^ Department of Energy and Materials Engineering, Dongguk University, Seoul, Korea

^10^ Department of Organic and Nano Engineering, Hangyang University, Seoul, Korea

†J. Kim and M.-G. Kim equally contributed to this work.

*To whom correspondence should be addressed. E-mail: **skpark@cau.ac.kr** (S.K.P)

Further details of materials and methods

Preparation of solution-processed semiconducting organic and carbon-based films.

Prior to deposition of semiconducting organic and carbon-based films, the substrates such as SiO_2_/Si and glass were cleaned in sonication bath of acetone, isopropanol for 10 min each, rinsing with isopropanol, and then blown dry using a N_2_ stream. Finally, O_2_ reactive ion etch (RIE) at 30 W for 1 min was done to remove residual organic contaminants from substrates.

1. C8-BTBT film. C8-BTBT solution was prepared by dissolving it in high-purity chloroform solvent at 2 wt% concentration. Prior to C8-BTBT film deposition, PMMA organic dielectric film was deposited on a SiO_2_/Si substrate so that larger grains of C8-BTBT crystalline domain can be achieved while also enhancing the wetting of C8-BTBT solution. A4 PMMA solution dissolved in anisole (Michrochem Co.) was spin-casted on a substrate at 4000 rpm for 40 sec, yielding about 200 nm-thick PMMA dielectric layer formation following baking at 200 °C for 5 mins. Then, C8-BTBT solution was spin-casted at 1000 rpm for 40 sec.

2. P3HT film. First, SiO_2_/Si substrate was dipped in 60 mM OTS (Sigma-Aldrich), diluted by high purity ethanol, for 30 min. Post-bake was done at 100 °C for 10 min to remove any solvent from a substrate. Hydrophobic surface formation was confirmed by the measurement of contact angle of water drop, about 109 degree, which consequently improves molecular ordering within P3HT polymer film for better device performance. Regioregular P3HT polymer (Sigma-Aldrich) was dissolved in high-purity chlorobenzene (anhydrous, Sigman-Aldrich) at 2 wt% concentration. Then, P3HT solution was placed on a hot plate for more than 30 mins while its temperature was set to 60 °C for complete dissolution. P3HT film was deposited by a spin-casting of its prepared solution on OTS-treated substrate at 1000 rpm for 40 sec. As-spun film was immediately thermally treated at 200 °C for 30 min. Note that all processes except OTS treatment were performed in N_2_-filled glovebox.

3. P-29-DPPDTSE and DPPT-TT films. Similar to P3HT film formation, high-performance diketopyrrolopyrrole-thieno[3,2-b]thiophene (DPPT-TT) (Polyera Inc.) and P-29-DPPDTSE polymers films are prepared by spin-casting of their solutions on aforementioned OTS-treated SiO_2_/Si substrate. Spin-casting of solutions was carried out at 2000 rpm from 0.2 wt% chloroform solutions. Then, films were annealed at 200 °C for 10 min. All procedures are done in N_2_-filled glove box.

4. PEDOT:PSS film. PEDOT:PSS solution (CLEVIOS PH 1000, Heraeus) was mixed with 5 wt% DMSO (anhydrous, ≥99.9%) and 1 wt% Zonyl FS-300 (Aldrich). The mixed solution was stirred for 2 hr before use. The solution was spin-casted on glass substrate at 2000 rpm for 60s. The film was annealed on a hot plate for 5 min at 120 °C under ambient condition.

5. Carbon-based films. The sorted poly(3-dodecylthiophene) (P3DDT)-wrapped CNT films was prepared with reported processes^1,2^. The sorted P3DDT-wrapped CNT solutions was prepared with suspension of HiPCO SWNT (5 mg) and the P3DDT (10 mg) in toluene (25 ml). The suspension was sonicated 500 W power for 30 min in dry ice acetone bath, and then was centrifuged at 4200 g for 150 min. The supernatant was collected and then spin-coated on top of O_2_ plasma treated 200 nm SiO_2_/Si at 5000 rpm for 40 drops.

Selective DUV photochemical deactivation

Selective DUV irradiation was made possible by a combination of chrome-patterned quartz photomask and a commercial ultraviolet cleaning system (UV-1 SAMCO Co. or EX-mini L12530 Hamamatsu Photonics K.K.) equipped with a low-pressure spiral-shaped mercury lamp or a RF discharge flat excimer lamp in a continuous N_2_ flow condition. Note that the low pressure spiral-shaped mercury lamp emits two main peaks at 253.7nm (90%) and 184.9nm (10%), with output intensity of 18~23 mW cm^-2^. The RF discharge lamp emits 172 nm with output intensity of 50 mW cm^-2^. The substrate was adhered to the quartz photomask by raising Z-stage following alignment of chrome-patterns of photomask relative to the devices using X-Y micromanipulator in a contact aligner. Chrome-pattern of photomask was designed in a way that it completely covers the channel region of devices during DUV irradiation. A few tiny drops of photoresist at the corners of substrate were sometimes necessary to ensure the firm attachment between the photomask and substrate during the entire DUV irradiation process.

It was found that a hard contact between the photomask and substrate was essential in transferring the fine patterns of photomask to the organic film due to the fringing effect. Thus, for fine patterning of organic films, 50 nm-sputtered chrome film on 1.5 mm-thick 2 cm × 2 cm quartz plate was patterned by a standard photolithography and subsequent wet etching processes. This small-sized photomask makes a close contact to the substrate by clamping them on four sides using a stainless steel office binder clips. Once the photomask with a substrate was loaded onto DUV irradiation system, 10 mins of N_2_ prepurge was allowed to make sure DUV irradiation chamber an inert gas atmosphere free of O_2_ and H_2_O. For selective deactivation of semiconducting organic films, DUV irradiation was continued for the desired time with each organic materials used in this study. Their DUV deactivation time was determined from a set of mask-free DUV irradiation experiment, which completely deactivates the functional organic films. So, typical DUV irradiation times for polymers and small-molecules in our experiment are fixed at 45 and 60 mins, respectively. Since DUV deactivation time depends on the energy and intensity of incident photons, 10 mins of the excimer lamp irradiation was sufficient to electrically isolate C8-BTBT devices as well as phochemically pattern the films. Gradual increase of temperature inside DUV chamber should be inevitable due to the direct heat radiation from low pressure mercury lamp. Temperature of photomask was ramped up to about 75 °C, measured by an infrared camera (InfraCAM, FLIR system), during 2 hrs continuous operation of DUV irradiation. Since this temperature is lower than typical annealing temperature of organic materials and they are placed even underneath the photomask, we exclude any significant thermal impact on DUV irradiation experiment.

Characterization.

DUV-irradiated as well as as-spun organic films was characterized by cross-polarized optical microscope. Morphological change of organic films including small-molecules and polymers upon DUV irradiation were evaluated by non-contact atomic force microscopy (XE-100, PSIA). Confocal Raman spectroscopy (300R, WiTec Co.) using 532 nm laser source and Fourier transform infrared spectroscopy (Nicolet 6700, Thermo Scientific Co.) were employed to assess the chemical bondings such as C-S, C-C, and C=C as well as intermolecular ordering within the organic films. For organic semiconductors of small molecule (C8BTBT) and polymers (P3HT, DPPT-TT, and P29-DPPDTSE), grazing-incidence wide-angle X-ray scattering (GIWAX) measurements were performed at the X9 beamline of the National Synchrotron Light Source at Brookhaven National Laboratory. The X-ray wavelength used was λ = 0.918 Å with an incidence of 0.1°. The scattering intensity was recorded on a two-dimensional image plate of 1,042 x 1,042 pixels, located at a distance of 216.18 mm from the sample center. The incidence angle was chosen as 0.1° with a beam size of 50 µm × 150 µm. The distance between the sample and the detector was calibrated using a silver behenate standard. The electrical characterization of fabricated TFTs and circuits was performed in air and dark conditions using Agilent 4155C semiconductor parameter analyzer.

Device fabrication.

For the fabrication of organic thin film transistors with a global back gate configuration, 4-inch, heavily-doped silicon substrate with a 200 nm thermally-grown oxide was used for all organic semiconducting films. For source and drain electrode formation, 50 nm-thick Au was thermally-evaporated through a metal shadow mask for top-contact devices and double layer of photoresists, GXR-601 (AZ Electronic Materials) and PMMA (Microchem Co.), for bottom-contact devices.

For the individually addressable devices, the gate electrode was patterned by conventional photolithography and wet etching following 50-nm-thick chrome sputtering on a glass substrate (Eagle 2000, Samsung Corning Co.). 35 nm-thick Al_2_O_3_ gate dielectric layer was deposited at 100 °C by atomic layer deposition (ALD) system using precursors of trimethyaluminum (TMA) and water for aluminum and oxygen, respectively. 50 nm-thick Au was thermally-evaporated on a double layer of photoresists and patterned by a liftoff process in acetone bath at 60 °C for source and drain electrodes. For flexible devices and circuits, a polyimide solution was spin-casted on a glass substrate and thermally-annealed at 300 °C for 1 hour to remove the solvent. Final thickness of polyimide film was estimated about 3 μm (measured by the surface profiler). Other fabrication steps are identical to those on a glass substrate as described above. For simplification of device measurement, the channel length and width were 50 µm and 1000 µm for all top-contact devices while 10 µm and 100 µm for bottom-contact ones, respectively, throughout this work.

**Further details of DUV photochemical deactivation**

**Morphological change of small molecule-based organic films following DUV irradiation**

Selective DUV irradiation using a chrome-patterned quartz mask enabled direct patterning of C8-BTBT/PMMA organic films as shown in Figure S1. CPOM images clearly exhibit DUV-irradiated and -protected regions within the film. Following DUV irradiation, birefringence of C8-BTBT organic crystalline region became nearly disappeared while rotating a polarizer by 15˚, which suggests the severe molecular disordering within the film by the bombardment of high energetic photons. In contrast, DUV-protected C8-BTBT/PMMA region seemed to retain its high crystallinity indicated by high birefringence from **Figure S1a** and **b**. Inset of Figure S2 indicates the surface morphology of as-spun C8-BTBT film, also exhibiting the step terrace of C8-BTBT molecular layers (~2.6 nm)^3^


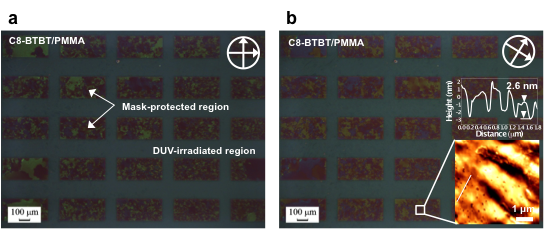


Figure S1. Selective DUV irradiation of C8-BTBT/PMMA organic film using a chrome-patterned quartz mask. a and b. CPOM images of C8-BTBT organic films while rotating a cross-polarizer by 15°. Mask-protected region still exhibits a strong birefringence while DUV-irradiated region became insensitive to the polarized light. The inset indicates AFM image and line scan of as-spun C8-BTBT organic film for its molecular step formation (~2.6 nm).

Figure S2 shows series of GIXRD pattern (top) and relevant CPOM images (bottom) of C8-BTBT small-molecule organic film as a function of DUV irradiation time. As DUV irradiation time increases, it is apparent that both out-of- and in-plane Bragg peaks became significantly diminished, suggesting gradual loss of inter-molecular ordering and disrupted molecular packing within the organic films. Not surprisingly, trend of a change in GIXRD patterns appeared to be qualitatively consistent with macroscopic observation of CPOM. One might notice that 60 min or longer DUV irradiation completely deactivates the organic semiconducting films, converting them into insulating one. It should be also noted from simultaneous disapperacne of out-of- and in-plance Bragg diffraction peaks from series of GIXRD patterns that well-ordered molecular packing of small-molcule organic films underwent isotropical collapse by an incidence of high energetic photons.


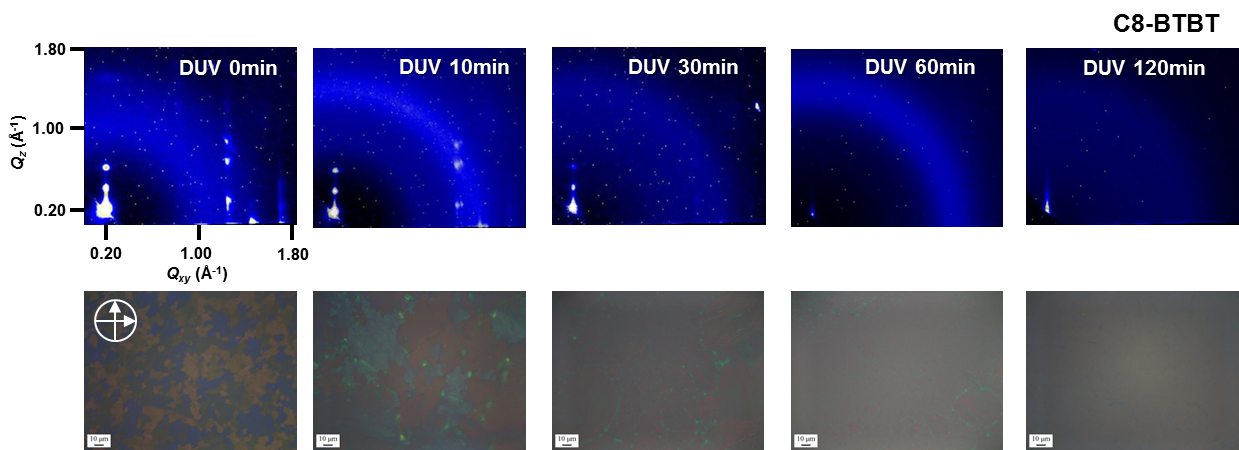


Figure S2. Gradual loss of inter-molecular ordering within small molecule-based organic films as a function of DUV irradiation time. CPOM images and GIXRD patterns of C8-BTBT small molecule organic films irradiated by DUV ray for 0, 10, 30, 60, and 120 min. Increased time of DUV irradiation on small molecule-based organic film gradually diminished their birefringence as a signature of intermoleuclar ordering.

Figure S3 a-c show series of GIXRD patterns in P3HT, P-29-DPPDTSE and DPPT-TT semiconducting polymers as a function of DUV irradiation time. Similar to small molecule-based organic films, Bragg diffraction peaks begins to diappear at longer DUV irradiation and almost invisible at 60 min of continuous DUV exposure. Thus, it is evident that DUV irradiation in an inert atmostphere photochemically transforms well-ordered semiconducting organic films including small molecule and polymers into structurally disordered state.


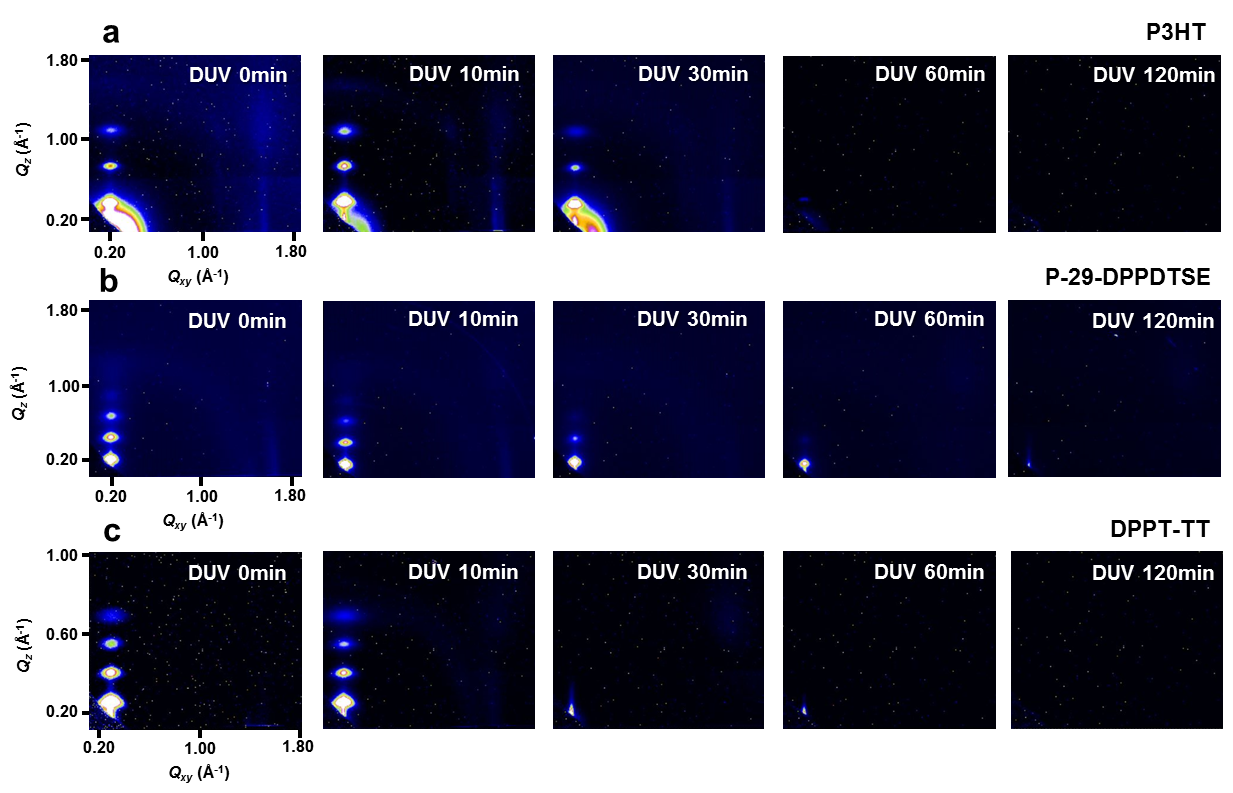


Figure S3. Gradual loss of inter-molecular ordering within polymer semiconducting organic films as a function of DUV irradiation time. GIXRD patterns of a. P3HT, b. P-29-DPPDTSE, and c. DPPT-TT polymer organic films irradiated by DUV ray for 0, 10, 30, 60, and 120 min.

Chemical bonding dissociation of polymer-based organic films by DUV irradiation

As discussed in the main text, it was found that high energetic photons induce chemical bonding dissociation as well as molecular disordering for small molecules and polymers. The Raman spectra (Figures S4a-d) with a wide range from 400 to 1800 cm^-1^ revealed significant reduction of characteristics peaks regarding chemical bonds within the organic films. Several peaks from as-spun P3HT were matched to C-S, C-C and C=C bonds according to literatures, although most of peaks from as-spun DPPT-TT and P-29-DPPDTSE remained unidentified. Disappearance of these peaks can be ascribed to dissociation of chemical bonds and complete loss of ordered region by virtue of DUV irradiation. In the case of graphene films, the excimer lamp with residual oxygen has been employed for patterning. Raman spectra in Figure S4d indicate the suppression of graphene 2D peak and significant increase of defect related D peak, with increased DUV irradiation time. Complete removal of graphene was found following the prolonged exposure of DUV excimer lamp (10 min), which supports the optical microscope image of periodically patterned graphene film in Figure 1c.


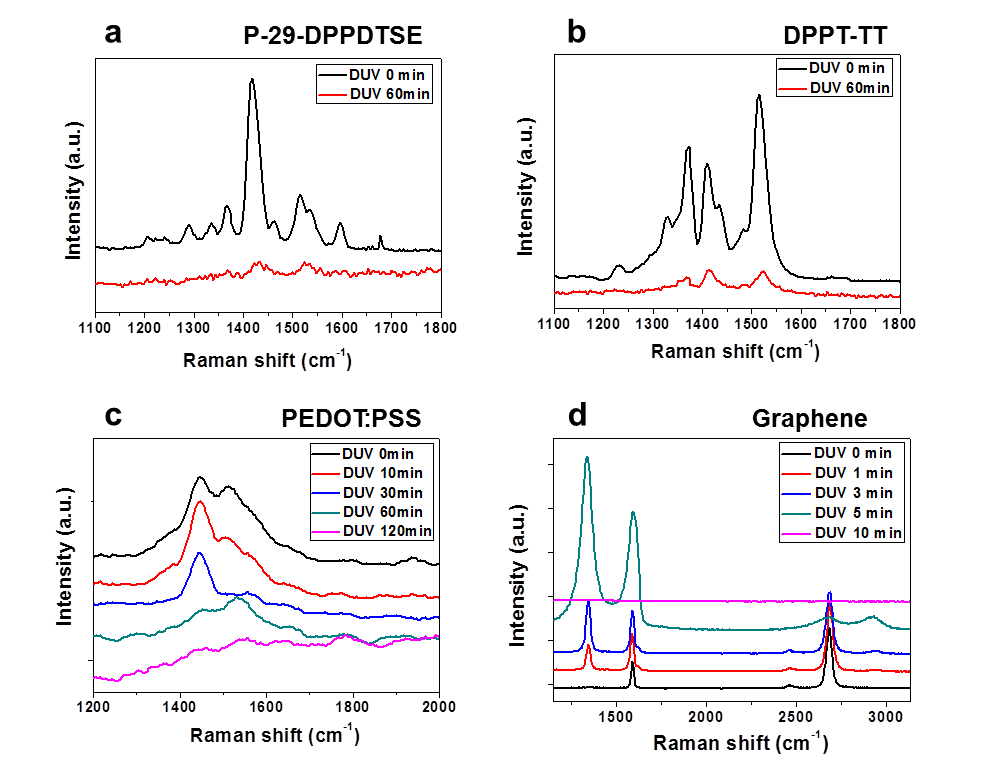


Figure S4. Chemical bond dissociation of small molecule, polymer and carbon-based organic films. Raman spectra of a. P-29-DPPDTSE and b. DPPT-TT, c. PEDOT:PSS and d. Graphene films as a function of DUV irradiation time. Intensity of characteristic organic-related peaks responsible for chemical bonds such as C-S, C-C, and C=C became significantly reduced by DUV irradiation. It suggests that incidence of high energetic photons ruptures the chemical bonds leading to intra-molecular dissociation as well as inter-molecular ordering.

Inferring from drastic change of internal molecular structure of DUV-irradiated organic films as discussed above, it is also probable that bombardment of high energetic photons into the soft material could induce the change of surface morphology of organic films used in this study. AFM surface scan of organic films before and after DUV irradiation exhibited loss of molecular steps as a proof for crystalline phase for small molecule organic films as shown in Figure S5 a.


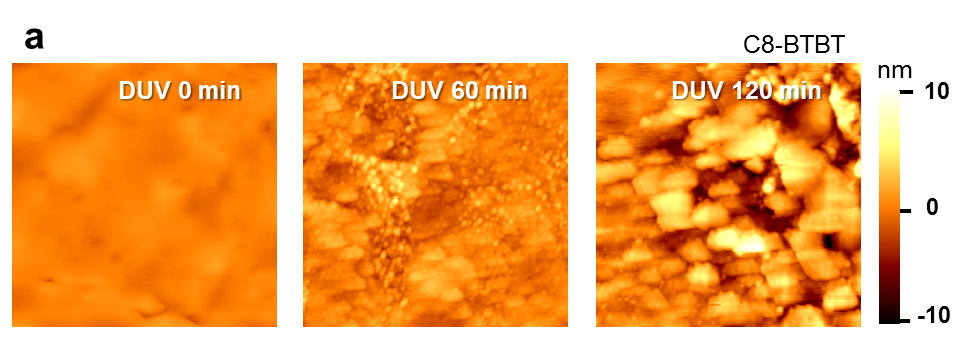


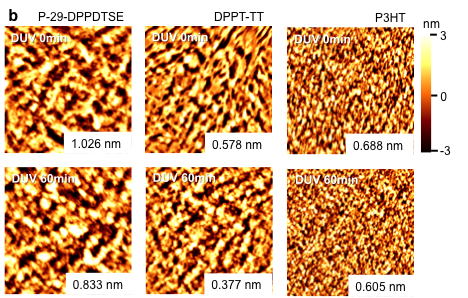


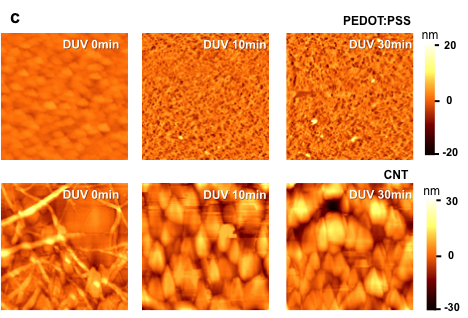


Figure S5. Morphological surface change of DUV-irradiated organic films. AFM surface scan of a. small molcule organic semiconductor (C8-BTBT), b. polymer semiconductor (P3HT, P-29-DPPDTSE, and DPPT-TT), and c. conductor (PEDOT:PSS) before and after DUV irradiation. All AFM surface scans are as large as 3 μm × 3 μm.

As shown in Figure S5 b, Slight reduction of surface roughness was found from DUV-irradiated polymer films, possibly resulting from their molecular flattened surface. Upon DUV-irradiation of organic conducting films for 10 min, disappearance of their characteristic surfaces such as aggregated surface took place as shown in Figure S5 c. Modification of surface change remained almost unchanged after 120 min DUV irradiation.

TFTs and circuits fabrication

Device isolation using selective DUV irradiation

Following fabrication of small molecule-based C8-BTBT OTFT array, chrome-patterned quartz photomask was aligned relative to the channel region and was brought into a hard contact so that chrome pattern locally blocks DUV irradiation. DUV irradiation for 45 min using this photomask defines the channel region of individual TFT in the array, which electrically isolates the adjacent devices as evident from CPOM images of Figure S6 a and b. White rectangles indicate the chrome pattern of photomask, intending to protect the active organic films at the channel region. It can be clearly seen from Figure S6 a and b that DUV-irradiated region exhibited significant loss of birefringence as a signature of molecular ordering. Thus, it is clear that chrome-pattern effectively blocks incoming DUV rays into the channel region of TFT while DUV irradiation took place elsewhere across the substrate.


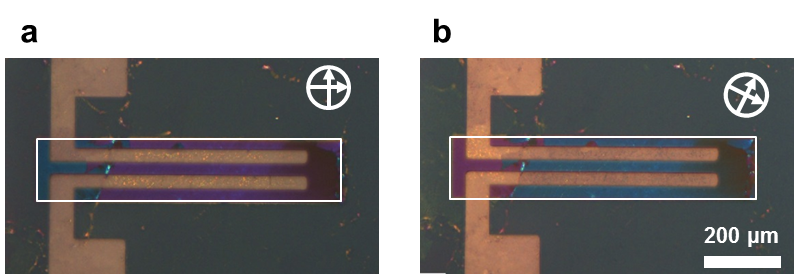


Figure S6. DUV isolation of C8-BTBT small molecule device. CPOM images of a. and b. top-contact C8-BTBT OTFTs on SiO_2_/Si substrate following spatially-selective DUV irradiation. White rectangles in CPOM images correspond to mask-protected active organics. Note that the channel regions between S/D electrodes remained intact during DUV irradiation.

**Figure S7** compares the transfer characteristics of P-29-DPPDTSE and DPPT-TT OTFTs, configured as top contact and global back gate, before and after selective DUV isolation. As discussed in main text, selective DUV irradiation enabled the active layer isolation of small molecule and polymer semiconducting organic films. For more detailed explanation regarding the device isolation, DPPT-TT as an example, off-state I_D_ from as-fabricated OTFT typically was measured as high as 10^-7^ A with an on-state I_DS_ of 10^-4^ A. I_GS_ also shows similar behavior as a function of V_GS_ due to considerable gate leakage current through SiO_2_ across whole conducting organic-covered substrate. DUV irradiation significantly reduces the dimension of gate-modulated charge accumulation region while also eliminating the incoming current flow from neighboring devices by the device isolation. As a result, I_GS_ appeared to diminish down to tens pA, quite comparable to physically-isolated devices, while following almost off-state I_DS_ at positive V_GS_ regime. So, the transfer curve of DPPT-TT OTFT in **Figure S7 b** shows the well-defined current modulation estimating 7×10^6^ on/off ratio improved from 10^3^ for the unpatterned devices.


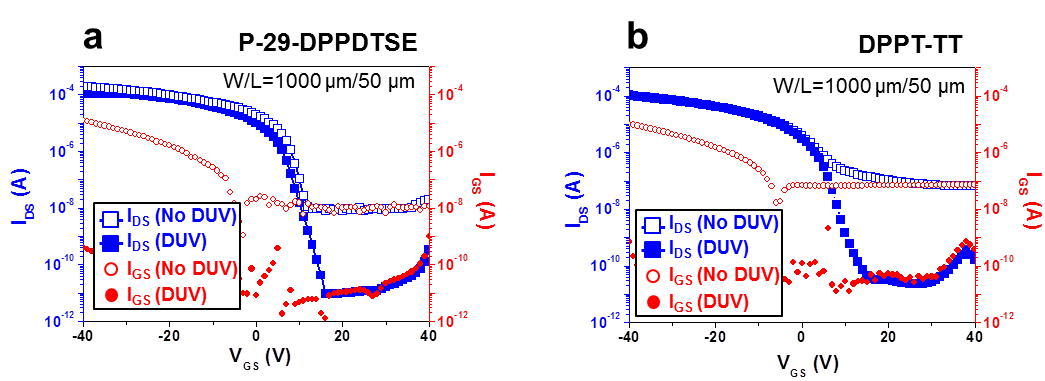


Figure S7. Electrical characteristics of OTFTs after selective DUV irradiation. Transfer curves of a. P-29-DPPDTSE and b. DPPT-TT OTFTs using top-contact on SiO_2_/n^++^-Si wafer (gate electrode).

Direct DUV exposure to the organic films without the photomask yielded the insulating organic films as shown in Figure S8. It is not surprising that DUV-exposed film became insulating. P-29-DPPDTSE film was used as an example in this study.


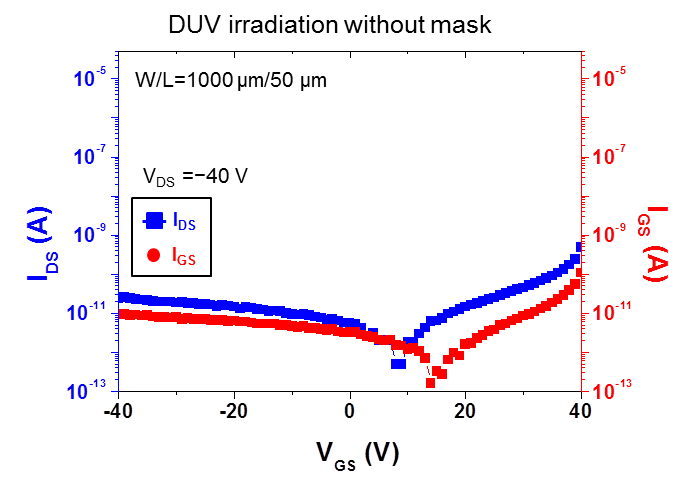


Figure S8. Electrical characteristics of P-29-DPPDTSE OTFT after mask-free DUV irradiation. Drain (blue) and gate (red) currents of P-29-DPPDTSE OTFT using top-contact on SiO_2_/n^++^-Si wafer (gate electrode).

Since the gate voltage modulation using a heavily-doped silicon substrate induces the charge accumulation of organic semiconductors across the substrate, relatively large I_DS_ at small V_DS_, can be found from the output curves of small molecule OTFTs, indicated by the red dashed circle in Figure S9 a. This unfavorable parasitic leakage current was minimized by the patterning of organic semiconductors using selective DUV irradiation as shown in Figure S9 b. Suppression of these parasitic leakage current in output curve of semiconducting polymer OTFTs can be also found from Figure S10.


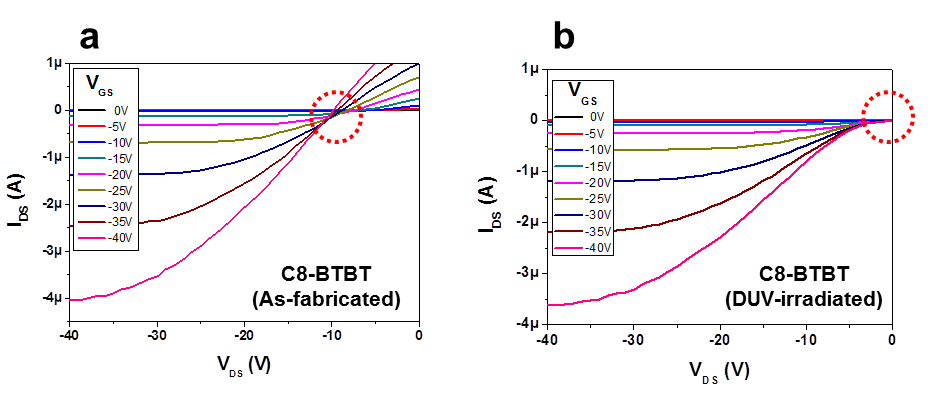


Figure S9. Electrical characteristics of small molecule-based OTFT after selective DUV irradiation. Output curves of C8-BTBT OTFTs a. before and b. after DUV isolation, respectively. Obviously, selective DUV irradiation minimized the parasitic leakage current at small V_DS_.


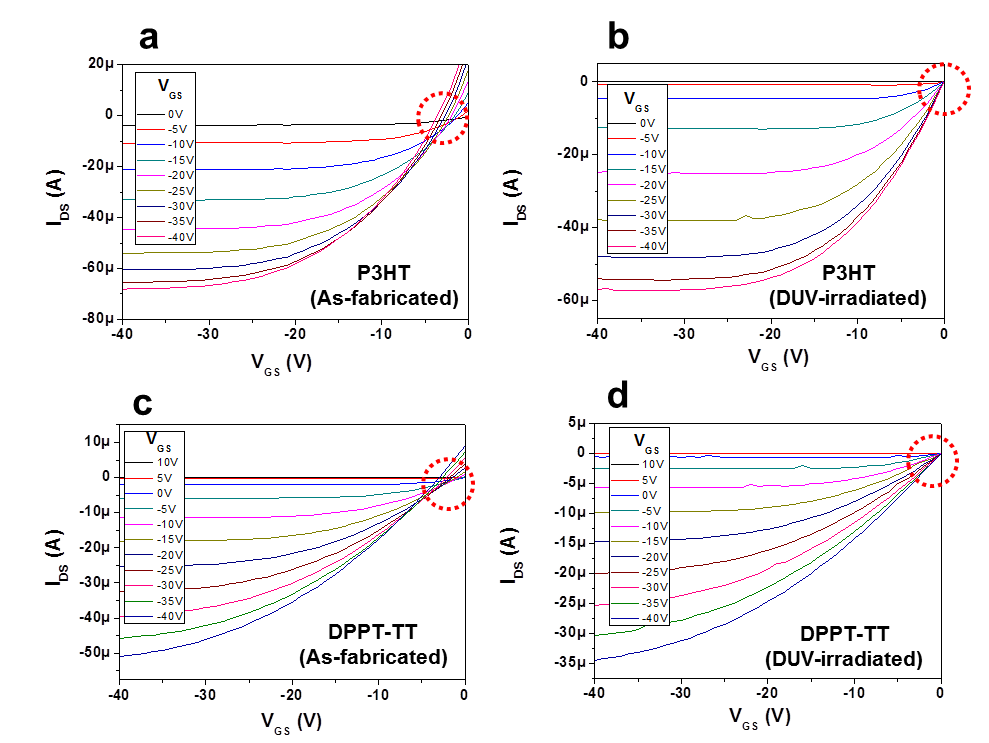


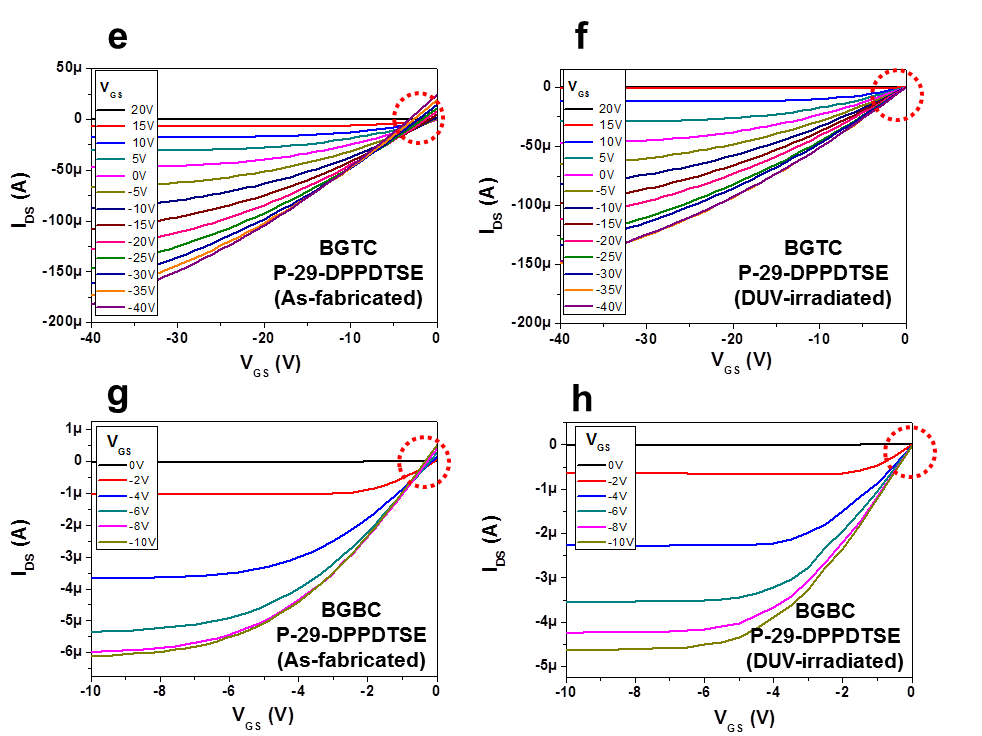


Figure S10. Electrical characteristics of semiconducting polymer-based OTFTs after selective DUV irradiation. Output curves of a. and b. P3HT, c. and d. DPPT-TT and e. and f. P-29-DPPDTSE OTFTs with top source/drain contact electrodes before and after DUV isolation, respectively, while g. and h. P-29-DPPDTSE OTFTs with bottom electrodes

We narrowed down the channel width (W) of P-29-DPPDTSE organic transistors down to 2 μm using the selective DUV irradiation. Gradual decrease of I_DS_ as a function of channel width seemed to be well matched with the theoretical prediction (I_DS_ ∝ W). This successful isolation suggests that further downscaling can be also possible. These results are summarized in Figure S11. Channel widths for the selected OTFTs are visible from their optical microscope images following DUV isolation.


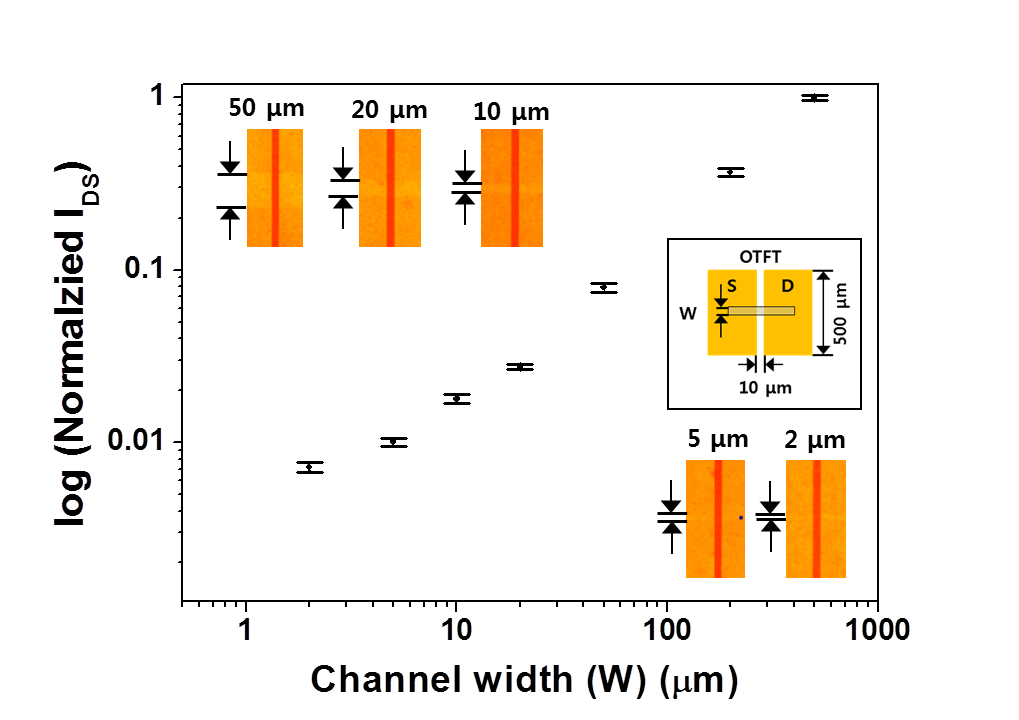


Figure S11. Relation of normalized I_DS_ of P-29-DPPDTSE OTFTs as a function of channel width (W). Gradual decrease of I_DS_ can be found with narrower channel width (W) in which their relation match well with the theoretical prediction (I_DS_ ∝ W). Note that the normalized I_DS_ corresponds to I_DS_ with DUV-patterned channel width (W) divided by I_DS_ of the unpatterned device (W=500 μm). The optical microscope images of DUV-patterned OTFTs with selected widths (2, 5, 10, 20, and 50 μm) are inserted.


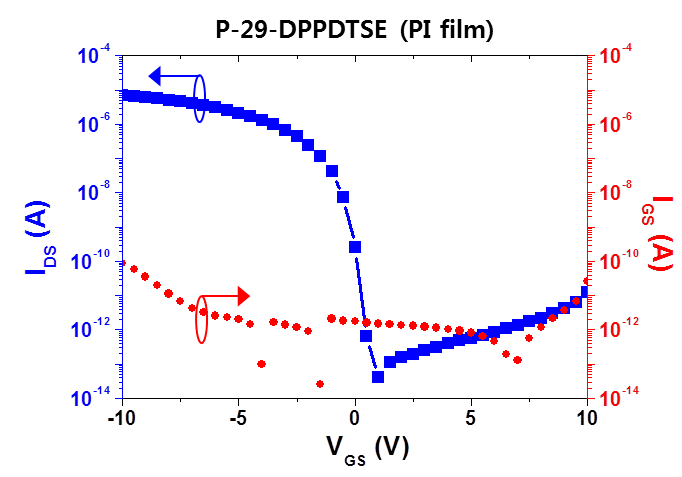


Figure S12. Electrical characteristics of OTFTs on 3-μm-thick flexible film after selective DUV irradiation. Transfer curves of P-29-DPPDTSE polymer films on a flexible film, exhibiting ~10^8^ modulation of drain current and a few pA of gate current.

Gain of P-29-DPPDTSE polymer-based inverter can be calculated as shown in Figure S13a. In addition to low static power consumption by DUV isolation, slight increase of gain from 2.24 to 2.63 was observed. The output current (I_out_) became noticeably decreased as an outcome of the reduced leakage current of patterned organic films as shown in Figure S13b.


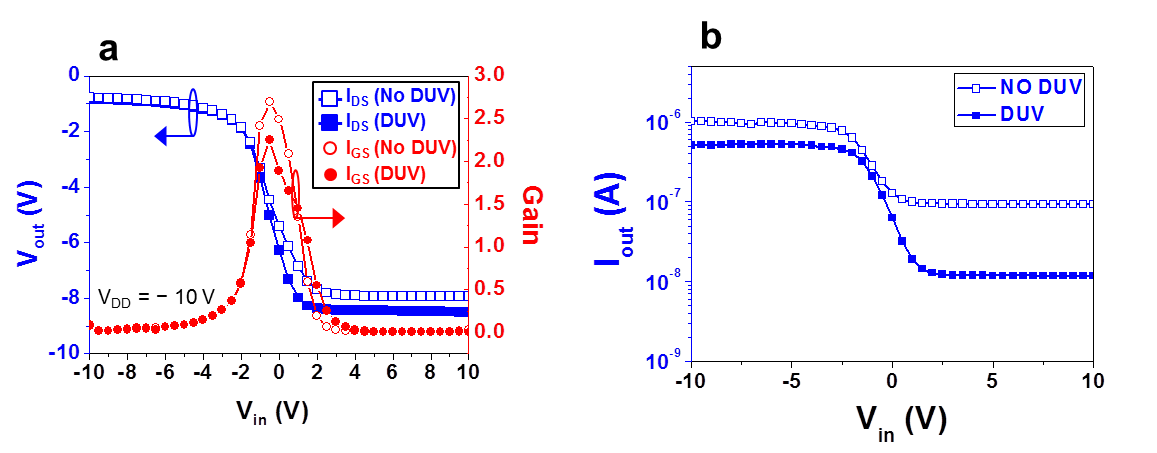


Figure S13. Gain of P-29-DPPDTSE OTFT-based inverter. a. Output voltage (V_out_) and b. output current (I_out_) characteristics of P-29-DPPDTSE organic inverter and after DUV device isolation

AIM-SPICE simulation was performed to demonstrate the effect of DUV patterning on P-29-DPPDTSE-based ring oscillator. Figure S14 compares the oscillating output currents of ring oscillators before and after DUV patterning, revealing the reduction of output current and consequential lower power consumption by about 20 %. In other words, unpatterned P-29-DPPDTSE ring oscillator exhibits oscillating waveforms with an average current of ~68.2 μA while the patterned P-29-DPPDTSE ring oscillator exhibits 20% lower average current of 55.1 μA. Since the same V_DD_ was applied, the transfer curves of discrete organic transistors with the same overlap dimensions and β ratio in Fig. 3c were used for the simulation for both ring oscillators.


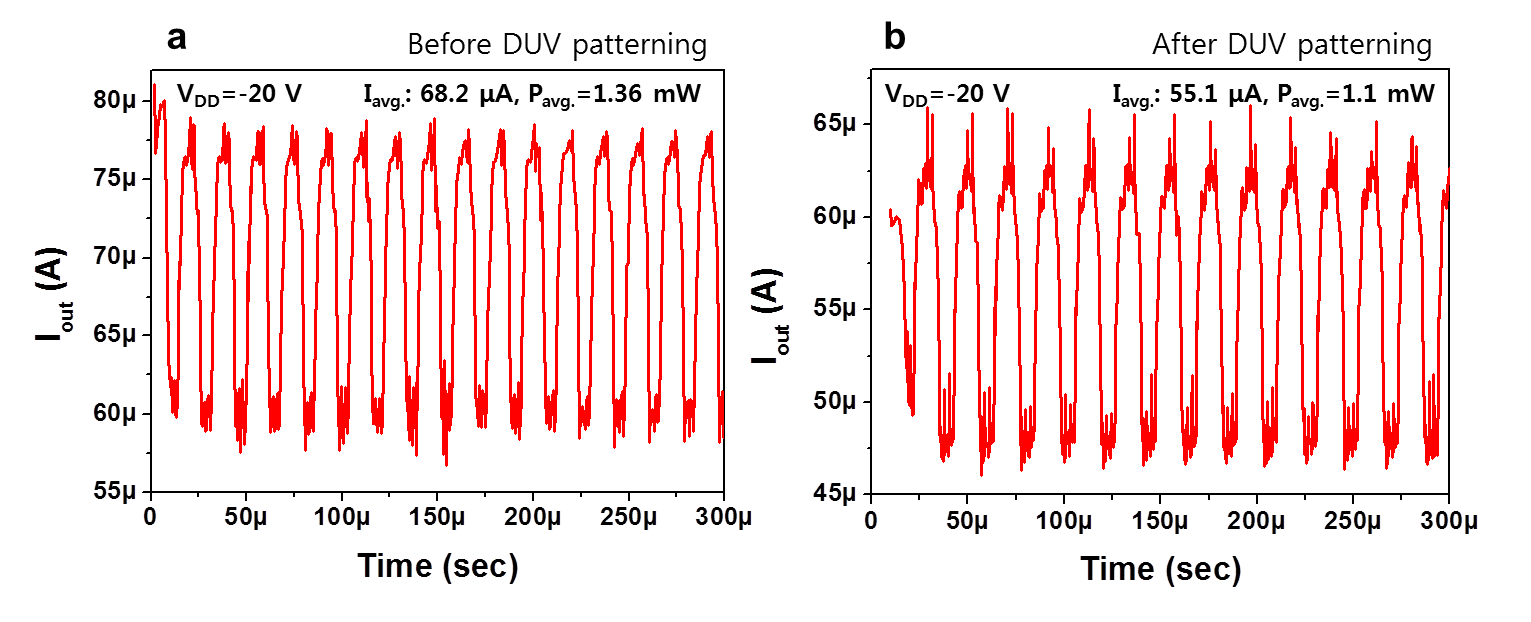


Figure S14. P-29-DPPDTSE organic ring oscillators. Output current (I_out_) characteristics of P-29-DPPDTSE organic ring oscillator a. before and b. after DUV device isolation.

Table S1. Summary of small molecule- and polymer-based OTFTs after selective DUV irradiation.


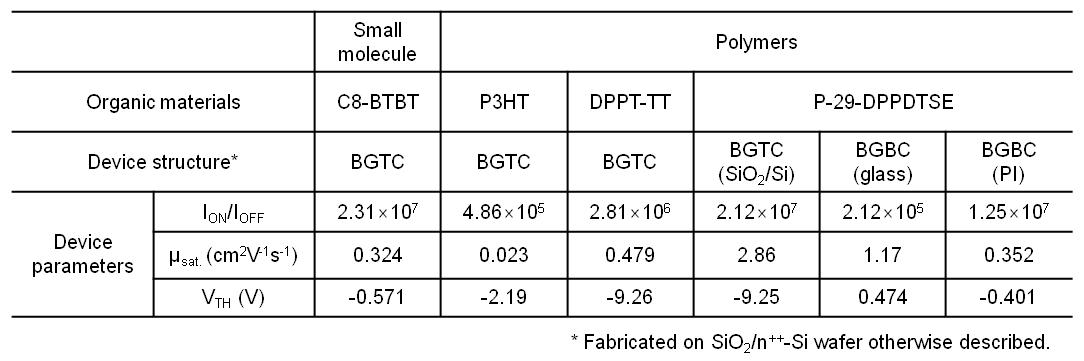


References

1. Lipomi, D. J. *et al.* Skin-like pressure and strain sensors based on transparent elastic films of carbon nanotubes. *Nat. Nanotechnol.* **6,** 788–792 (2011).

2. Lee, H. W. *et al.* Selective dispersion of high purity semiconducting single-walled carbon nanotubes with regioregular poly(3-alkylthiophene)s. *Nat. Commun.* **2,** 541 (2011).

3. Minemawari, H. *et al.* Inkjet printing of single-crystal films. *Nature* **475,** 364–7 (2011).
